# Effects of sodium roasting on the leaching rate of boron-bearing tailings and its mechanism analysis

**DOI:** 10.1098/rsos.172342

**Published:** 2018-08-08

**Authors:** Chengxi Zou, Zhenyu Tang, Wei Xie, Hanguang Fu, Jiacai Kuang, Yingjun Deng, Taishan Cao

**Affiliations:** 1 Key Laboratory of Lightweight and Reliability Technology for Engineering Vehicle, Education Department of Hunan Province, Changsha University of Science and Technology, Changsha 410114, People's Republic of China; 2 College of Materials Science and Engineering, Beijing University of Technology, Beijing 100124, People's Republic of China

**Keywords:** boron-bearing tailings, sodium roasting, leaching rate, sodium borate

## Abstract

The study reported was intended to improve the leaching rate of boron-bearing tailings, using a method of sodium roasting that uses boron-bearing tailings as the raw material and Na_2_CO_3_ as the sodium agent. The effects of the roasting temperature and Na_2_CO_3_ amount on the leaching rate of boron-bearing tailings are mainly evaluated. The morphology and composition of the samples after sodium roasting are analysed by scanning electron microscopy and X-ray diffraction. The results show that sodium roasting can significantly improve the leaching rate of boron-bearing tailings. Under the optimal conditions where roasting temperature is 950°C, Na_2_CO_3_ amount is five times the theoretical amount and roasting time is 2 h, the leaching rate of boron-bearing tailings is up to 86.78%. Based on the analysis of the characterization results and the mechanism analysis of the sodium roasting process, the main reason for the increase of leaching rate is the reaction between Na_2_O produced by the decomposition of Na_2_CO_3_ and the boron in boron-bearing tailings resulting in soluble sodium borate. The results provide a scientific basis for the efficient comprehensive use of boron-bearing tailings.

## 1. Introduction

As an important ore resource, boron deposit is the basic raw material for the production of borax [1,2], boric acid [3,4] and a series of other boron and boride products [5,6], which are widely used in fields such as chemicals, metallurgy, building materials, electrical appliances, machinery, agriculture, nuclear industry and medicine [7–10]. The global boron ore resources are relatively concentrated, and there are few countries with boron resources. The global boron ore reserves are about 170 million tons, of which Turkey, the United States, Russia and China account for about 97% of the global reserves. Some researchers have attempted to study various boron products in recent years. Özdemir [11] proposed a two-step process for boron recovery from borax sludge. Celik [12] investigated the effect of boron waste on the physico-mechanical properties of ceramic wall tiles by replacing marble in suitable amounts to demonstrate its suitability for industrial production. However, China is rich in boron ore resources, with the total reserves of boron ore resources ranking the fourth in the world, which are mainly distributed in Liaoning, Jilin, Tibet and Qinghai [13]. The vast majority of boron ore processing methods use ascharite as the raw material to produce borax and boric acid which are used as basic inorganic chemical raw materials and can be further processed into other boron compounds [14,15]. Previous studies have shown that the leaching rate of boron ore determines the complexity of preparing borax and boric acid, and that how to improve the leaching rate of boron ore therefore becomes a key scientific problem in the deep processing of boron ore resources [16–19]. After 60 years of deploitation and use, the grade of China's ascharite ore has been reduced, and the reserves are less than 2 million tons, with many mines depleted, so the reserves cannot meet the development needs of modern boron industry any more, creating a very prominent supply and demand contradiction. The development and use of other boron ore resources, as the alternative of ascharite, has become a top priority. In addition, as a result of boron removal in paigeite, the paigeite in the Kuandian area, Dandong, Liaoning produces a large amount of boron-bearing tailings with a leaching rate of only 45–55%. Furthermore, because of their low leaching rate, boron-bearing tailings cannot be used as raw materials for borax preparation and are difficult to be processed and used. Therefore, it is necessary and urgent to carry out research on the improvement of the leaching rate of boron-bearing tailings.

Sodium roasting technology is a method [20] that adds sodium agents (for example, sodium carbonate, sodium sulfate and sodium hydroxide) in mineral raw materials, and uses the characteristics of sodium agents which will be decomposed into Na_2_O. Na_2_O is of higher leaching rate and liable to combine with other elements to form soluble sodium salts under a certain temperature and atmosphere condition, which can also destroy the structure of the minerals to facilitate the further leaching of target elements. Liu [21], Li [22] and Jiang [23] attempted to improve the leaching rate of boron ores using a sodium roasting method with paigeite ores, ludwigite ores and boron concentrate ores as the raw materials and Na_2_CO_3_ as the sodium agent, and the leaching rate of boron was successfully improved to 93.30%, 72.10% and 91.05%, respectively, providing a favourable condition for the subsequent leaching of boron. Compared with the above raw materials, boron-bearing tailings are of low grade and complex chemical composition, which causes difficulties in leaching boron and comprehensive use. Currently, there are few studies on the leaching rate mechanism of low-grade boron-bearing tailings. Therefore, it is of great significance to improve the leaching rate of boron-bearing tailings. Our previous paper [24] mainly studied the influence of sodium roasting on boron recovery, while this paper mainly focuses on the influence of sodium roasting on the leaching rate of boron. The preliminary research is of guiding significance to the later research.

Based on the studies above, this paper aims to purify low-grade boron-bearing tailings and increase the overall utilization rate of boron resources. This paper takes boron-bearing tailings as the subject and Na_2_CO_3_ as the sodium agent, and mixes boron-bearing tailings with Na_2_CO_3_ for hybrid roasting. The main phases of the boron-bearing tailings include serpentine, talc, tremolite, chlorite, szaibelyite, muscovite, forsterite, quartz and magnetite. The purpose of the paper is to investigate the effects of roasting temperature, Na_2_CO_3_ amount and roasting time on the leaching rate of boron-bearing tailings in the process of sodium roasting. Preliminary analysis is also conducted on the mechanism of the method that sodium roasting can improve the leaching rate of boron-bearing tailings, so as to provide a scientific basis for the efficient comprehensive use of boron-bearing tailings.

## 2. Experimental

### 2.1. Chemicals and materials

**Figure 1.**
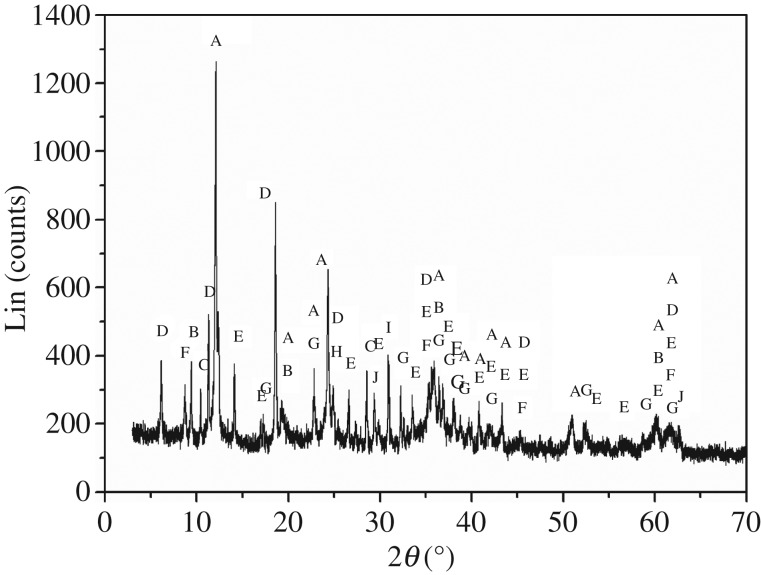
XRD pattern of Kuandian ores. (A) Mg_6_[Si_4_O_10_](OH)_8_; (B) Mg_3_[Si_4_O_10_](OH)_2_; (C) Ca_2_Mg_5_Si_8_O_22_(OH)_2_; (D) Mg_3_[Si_4_O_10_](OH)_2_Mg_3_(OH)_6_; (E) Mg_2_(OH)[B_2_O_4_(OH)]; (F) KAl_2_(AlSi_3_O_10_)(OH)_2_; (G) Mg_2_SiO_4_; (H) SiO_2_; (I) CaMg(CO_3_)_2_; (J) Fe_3_O_4_.

The boron-bearing tailings (Kuandian ores) were obtained from Kuandian, Liaoning province, China, with chemical compositions presented in table 1 and X-ray diffraction (XRD) pattern in figure 1. As can be seen from figure 1, the phases of boron-bearing tailings mainly include serpentine (Mg_6_[Si_4_O_10_](OH)_8_), talc (Mg_3_[Si_4_O_10_](OH)_2_), tremolite (Ca_2_Mg_5_Si_8_O_22_(OH)_2_), chlorite (Mg_3_[Si_4_O_10_](OH)_2_Mg_3_(OH)_6_), szaibelyite (Mg_2_(OH)[B_2_O_4_(OH)]), muscovite (KAl_2_(AlSi_3_O_10_)(OH)_2_), forsterite (Mg_2_SiO_4_), quartz (SiO_2_), dolomite (CaMg(CO_3_)_2_) and magnetite (Fe_3_O_4_). Among them, szaibelyite (Mg_6_[Si_4_O_10_](OH)_8_) is the main boron-bearing phase of the raw material tailings. The other chemicals used in the experiments, including sodium carbonate (Na_2_CO_3_), hydrochloric acid (HCl), calcium carbonate (CaCO_3_) and sodium hydroxide (NaOH), were of analytical grade and purchased from Hengyang Kaixin Chemical Reagent Co. Ltd. Deionized water was also used during the experiments.

**Table 1.** Chemical compositions of Kuandian ores.

directory	B_2_O_3_	Na_2_O	Al_2_O_3_	CaO	MgO	SiO_2_	Fe_2_O_3_	K_2_O
content (wt%)	9.98	0.04	1.36	2.28	32.43	28.41	4.88	0.03

### 2.2. Experimental procedure

The amount of Na_2_CO_3_ was defined as enough for the full conversion of B_2_O_3_ in the tailings to Na_2_B_4_O_7_ in stoichiometric ratios as the theoretical amount, and the theoretical mass of Na_2_CO_3_ was calculated as 1.13 g. To improve homogeneity, the ores were pulverized to 200 mesh, with 15 g evenly ground Kuandian ores and different theoretical amounts (one to six times the theoretical mass) of Na_2_CO_3_ fully mixed. Thereafter, the mixture was placed into a corundum crucible and was roasted in a heat treatment furnace at the preset temperature [25] (700–950°C). Heating was suspended when the default time was reached. The roasted ore samples were pulverized by a micro-pulverizer, and ground subject to a 200 mesh standard sieve. Afterwards, 4 g roasted ore samples were ground and sieved, and were mixed with 20 ml deionized water. Whereafter, the mixture was reacted in a micro-high-pressure reactor at 150°C for 6 h, and then it was cooled to room temperature and filtered. The residues were washed by deionized water until they became neutral, and were dried and accurately weighed for backup use. Then, 1 g dried, ground and sieved water leaching residues were taken out and placed in a 250 ml Erlenmeyer flask. Subsequently, 20 ml (1 + 1) hydrochloric acid (1 : 1 mixture of concentrated HCl and deionized water) was added, and the mixture was heated in an electronic attemperation electric jacket, allowing it to undergo reflux after slight boiling for 0.5 h. The leaching rate of boron-bearing tailings was defined as the percentage of B_2_O_3_ leached from the system, and it was evaluated by the pressurized water leaching rate of B_2_O_3_. The content of B_2_O_3_ in the ore samples and water leaching residues was determined according to GB3447.3-1982 [26]. The pressurized water leaching rate of the ore samples was calculated by the following formula:

2.1 $$X=\left(1-\frac{m_{2}\times W_{2}}{m_{1}\times W_{1}}\right)\times 100\text{\%},$$

where *X* is the leaching rate of ore samples, %; *m*_1_ is the mass of ore samples, g; *m*_2_ is the mass of water leaching residues, g; *W*_1_ is the content of B_2_O_3_ in ore samples, %; and *W*_2_ is the content of B_2_O_3_ in water leaching residues, %.

### 2.3. Characterization

The microstructure of the samples was observed by a MIRA3 TESCAN scanning electron microscope. The XRD patterns were recorded with a D/max2550VB+ X-ray diffractometer using Cu Ka radiation (*k* = 0.154178 nm) with 40 kV scanning voltage, 40 mA scanning current and its scanning ranging from 3° to 70°. Thermogravimetric-differential thermal analysis (TG-DTA) of the roasting process of boron-bearing tailings was carried out with an STA409PC comprehensive thermal analyser. Test conditions are as follows: the temperature rise rate is 10° min^−1^; the range is 0–1000°C; and the reaction occurs in an oxygen atmosphere.

## 3. Results and discussion

**Figure 2.**
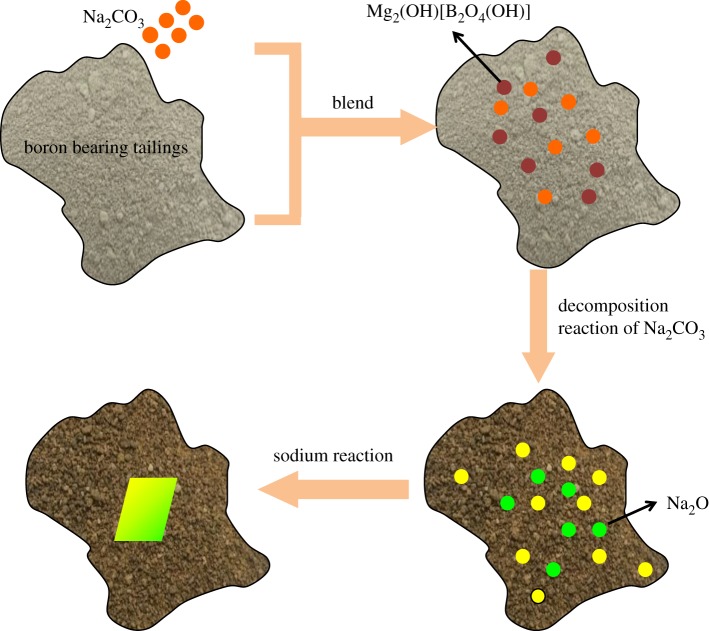
The process of sodium roasting reaction.

This paper conducts a preliminary analysis of the sodium roasting process, as shown in figure 2. The boron in boron-bearing tailings is mainly in the form of szaibelyite (Mg_2_(OH)[B_2_O_4_(OH)]). After it is mixed with appropriate amount of Na_2_CO_3_, the mixture will undergo chemical reaction at a certain roasting temperature. Primarily, the phase of szaibelyite is dehydrated during the roasted stage. Subsequently, with the roasting time prolonged, the decomposition of Na_2_CO_3_ starts in the case of high temperature calcination, and then the sodium reaction begins. Finally, the boron is in the form of sodium borate that is easy to be leached.

The temperature of sodium roasting may affect the occurrence of sodium reaction and its degree of reaction. The mixtures of tailings and sodium carbonate were roasted at different temperature (from 700°C to 950°C). The amount of sodium carbonate (5.65 g) was controlled at five times the theoretical amount, with 2 h roasting time. The results of the leaching rate are shown in table 2. As can be seen from table 2, the leaching rate of the Kuandian ores increases with the increase of roasting temperature. When the roasting temperature is 950°C, the leaching rate of Kuandian ores reaches 86.78%. Taking the energy consumption into account, experiments will not be conducted at higher temperature.

**Table 2.** The leaching rate of samples roasted at different calcination temperatures.

temperature (°C)	700	750	800	850	900	950
leaching rate (%)	58.78	65.82	69.39	78.08	80.46	86.78

Similarly, the amount of sodium carbonate used may affect the substances of boron-bearing tailings involved in the sodium reaction. The results of the leaching rate are shown in table 3, when the roasting temperature is controlled as 950°C and for a roasting time of 2 h. The amount of Na_2_CO_3_ used is one to six times the theoretical amount (1.13–6.78 g). As can be seen from table 3, during the process where the amount of sodium carbonate increases from one times the theoretical amount to five times, the leaching rate increases with the amount of sodium carbonate increasing. When the amount used is five times the theoretical amount, the leaching rate reaches the highest level (86.78%). When the amount of sodium carbonate increases to six times the theoretical amount, the leaching rate decreases slightly compared to that of five times.

**Table 3.** The leaching rate of samples with different amounts of Na_2_CO_3._

masses of Na_2_CO_3_ (g)	1.13 g	2.26 g	3.39 g	4.52 g	5.65 g	6.78 g
leaching rate (%)	18.58	47.36	62.90	75.18	86.78	76.21

The roasting time may affect the degree of sodium roasting. The results of the leaching rate are shown in table 4, when the roasting temperature is controlled as 950°C and the amount of sodium carbonate is five times the theoretical amount. The roasting time varies from 0.5 h to 3 h. As can be seen from table 4, during the process where the sodium roasting time increases from 0.5 h to 2 h, the leaching rate increases with the increase of roasting time, and it reaches the highest level of 86.78% at 2 h. During the process of roasting time increasing from 2 h to 3 h, the leaching rate decreases slightly with the increase of time.

**Table 4.** The leaching rate of samples roasted for different times.

roasting time (h)	0.5	1	1.5	2	2.5	3
leaching rate (%)	63.44	73.06	82.03	86.78	76.14	70.80

**Figure 3.**
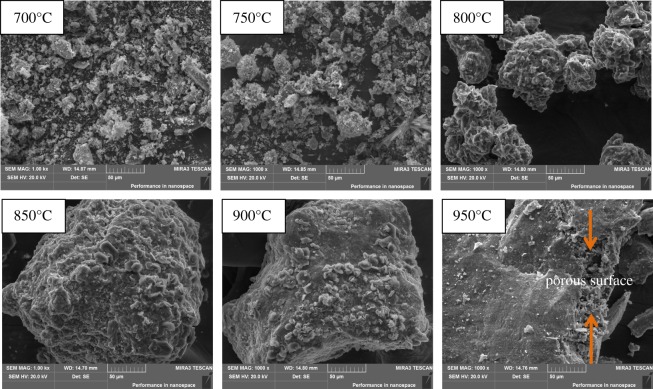
SEM images of samples roasted at different temperatures.

For the investigation of the microstructural changes of the ore samples during the roasting of the mixture of boron-bearing tailings and sodium carbonate, scanning electron microscopy (SEM) analysis has been conducted on the ore samples roasted at different temperature when the added amount of sodium carbonate is controlled as five times the theoretical amount and roasting time is 2 h. Figure 3 shows SEM images (magnified 1000 times) of the samples after being roasted at different sodium roasting temperature. As shown in figure 3, with the increase of the sodium roasting temperature, overall the granularity and bulk volume of the ore samples increase gradually, and the structure also gradually becomes clearer and more regular. There are some pores appearing in the samples after roasting at 950°C, which are mainly caused by the large amount of CO_2_ effused during the process of sodium reaction. These pores increase the specific surface area of the sample, so as to improve its reactivity and the leaching of the soluble sodium borate, which is beneficial to improve the leaching rate of the tailings.

**Figure 4.**
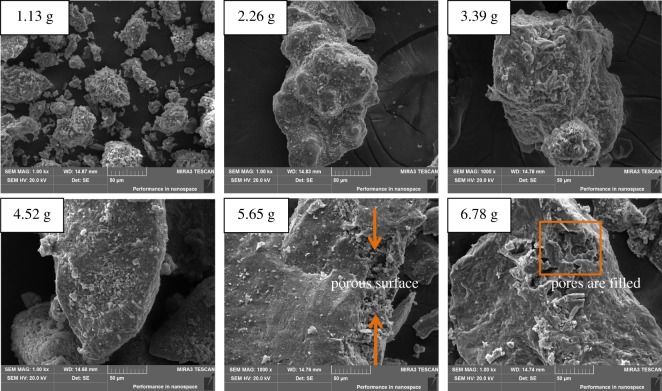
SEM images of samples with different amounts of Na_2_CO_3_.

Likewise, in order to study the effects of sodium carbonate amount on the leaching rate of boron-bearing tailings, SEM analysis is conducted on the ore samples roasted with different sodium carbonate amount. In this part, the roasting temperature is controlled as 950°C and the roasting time is 2 h. Figure 4 shows SEM images (magnified 1000 times) of the ore samples after being roasted with different sodium carbonate amounts. As shown in figure 4, with the increase of the sodium carbonate amount, overall the granularity and bulk volume of the ore samples increase gradually, and the structure also becomes clearer and more regular. There are some pores appearing in the samples when the sodium carbonate is five times the theoretical amount, which are mainly caused by the large amount of CO_2_ produced during the process of sodium reaction. These pores increase the specific surface area of the ore sample, which is beneficial to improve the leaching rate of the samples. However, when the added amount of sodium carbonate is six times the theoretical amount, excessive sodium carbonate is easily wrapped on the surface of the ore samples, so that the pores formed by the reaction are filled, reducing the specific surface area of the ore samples and hindering the leaching of sodium borate during the further reaction. Therefore, the leaching rate of the ore samples will decrease when the added sodium carbonate amount is six times the theoretical amount.

**Figure 5.**
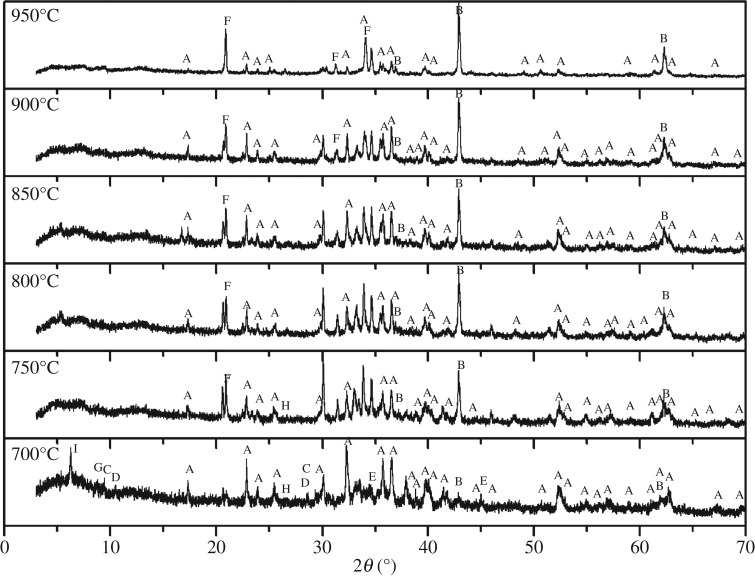
XRD patterns of samples roasted at different temperatures. (A) Mg_2_SiO_4_; (B) MgO; (C) Mg_3_[Si_4_O_10_](OH)_2_; (D) Ca_2_Mg_5_Si_8_O_22_(OH)_2_; (E) Mg_2_B_2_O_5_; (F) Na_2_B_4_O_7_; (G) KAl_2_(AlSi_3_O_10_)(OH)_2_; (H) SiO_2_; (I) Mg_3_[Si_4_O_10_](OH)_2_Mg_3_(OH)_6_.

With the aim to further study the phase change of the ore samples during the roasting process, six samples obtained at different roasting temperature were subjected to XRD analysis. As shown in figure 5, the main phases in the figure are forsterite (Mg_2_SiO_4_), magnesium oxide (MgO), talc (Mg_3_[Si_4_O_10_](OH)_2_), tremolite (Ca_2_Mg_5_Si_8_O_22_(OH)_2_), chalcone (Mg_2_B_2_O_5_), sodium tetraborate (Na_2_B_4_O_7_) muscovite (KAl_2_(AlSi_3_O_10_)(OH)_2_), quartz (SiO_2_), and chlorite (Mg_3_[Si_4_O_10_](OH)_2_Mg_3_(OH)_6_). The characteristic peak of Mg_2_B_2_O_5_ appears in the samples at 700°C. Nevertheless, there is no characteristic peak of sodium borate, because the decomposition temperature of Na_2_CO_3_ is 744°C, which means it fails to decompose and cannot react with Mg_2_B_2_O_5_ to produce sodium borate at 700°C. Therefore, the leaching rate at 700°C is too low. When the roasting temperature is above 750°C, the characteristic peak of Mg_2_B_2_O_5_ disappears and the characteristic peak of Na_2_B_4_O_7_ appears. It shows that Na_2_CO_3_ starts to decompose to Na_2_O which reacts with Mg_2_B_2_O_5_ to form Na_2_B_4_O_7_. At this temperature, sodium reaction starts and the leaching rate improves obviously. When the roasting temperature rises above 850°C, the characteristic peak of Na_2_B_4_O_7_ obviously increases, and so does the diffraction peak intensity. That is because the melting point of Na_2_CO_3_ is 851°C. When the temperature is over 850°C, Na_2_CO_3_ is in molten state as it is close to or beyond its melting point. The sodium roasting process is thus transformed from solid–solid reaction to solid–liquid reaction, which therefore makes the Na_2_CO_3_ contact with the tailings more fully and accelerates the reaction of Na_2_O with boron, so the leaching rate will increase with the further increase of the temperature.

**Figure 6.**
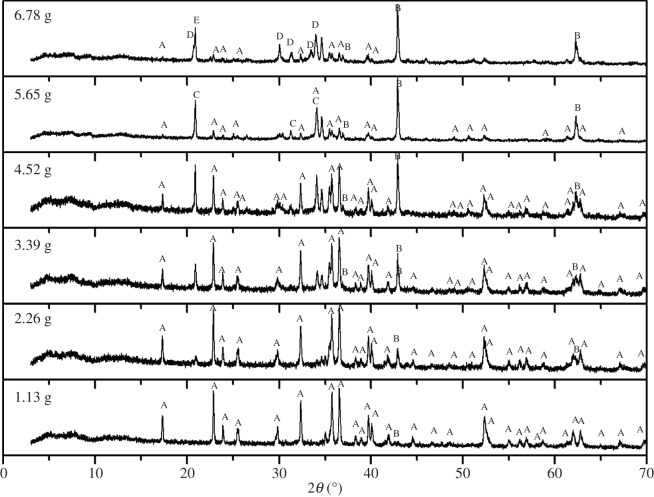
XRD patterns of sodium samples with different amounts of Na_2_CO_3_. (A) Mg_2_SiO_4_; (B) MgO; (C) Na_2_B_4_O_7_; (D) Na_4_Mg_2_Si_3_O_10_; (E) NaBO_2_.

The XRD patterns of the samples roasted with different sodium carbonate amounts are shown in figure 6. The main phases in the figure are forsterite (Mg_2_SiO_4_), magnesium oxide (MgO), sodium tetraborate (Na_2_B_4_O_7_), silicate (Na_4_Mg_2_Si_3_O_10_) and sodium metaborate (NaBO_2_). As shown in figure 6, when the amount of sodium carbonate is one to four times the theoretical amount, there are mainly characteristic peaks of Mg_2_SiO_4_ phase and MgO phase but no characteristic peak of soluble sodium borate, which is mainly because Na_2_CO_3_ decomposes to Na_2_O that reacts to quartz (SiO_2_) in the sample. Under these conditions (one to four times), the leaching rate is relatively low. When the amount of sodium carbonate increases to five times the theoretical amount, the characteristic peak of Na_2_B_4_O_7_ appears. The reason lies in that Na_2_O (decomposed by Na_2_CO_3_) begins to react with Mg_2_B_2_O_5_, and thus Na_2_B_4_O_7_ is produced which is easier to be leached, so the leaching rate reaches the highest level under such condition [27]. When the amount of sodium carbonate increases to six times the theoretical amount, the characteristic peaks of Na_4_Mg_2_Si_3_O_10_ and NaBO_2_ appear, which are caused by the reaction of Na_2_O (produced by excessive Na_2_CO_3_) with Mg_2_SiO_4_ and Mg_2_B_2_O_5_, respectively. As the melting point of Mg_2_SiO_4_ is high, it is easy to precipitate crystals. When the melt begins to cool, Mg_2_SiO_4_ is precipitated first and the tiny grains formed are evenly distributed in the melt, which plays the role of crystallization induction in the crystallization of sodium borate and promotes the crystallization of boron [28]. When the Na_2_O produced by excessive Na_2_CO_3_ reacts with Mg_2_SiO_4_ to form Na_4_Mg_2_Si_3_O_10_, Mg_2_SiO_4_ crystal grains are decreased, which weakens the effect of crystallization induction and decreases the crystallization of sodium borate and therefore results in the decrease of leaching rate.

XRD analysis was performed on the samples roasted for 0.5 h, which mainly contain Mg_2_SiO_4_ and MgO and slight characteristic peaks of Na_2_B_4_O_7_. As the roasting time is prolonged, Na_2_CO_3_ can contact and react with Mg_2_B_2_O_5_ in the tailings more fully, and its ability to destroy the crystal structure of the samples is increased, which increases the amount of Na_2_B_4_O_7_ and improves the leaching rate of the samples. When the roasting time is prolonged further, partial sodium borate produced is enclosed due to the rich content of silicate minerals in the tailings, which thus impedes the leaching of boron and then results in a slight decline of the leaching rate of boron-bearing tailings.

**Figure 7.**
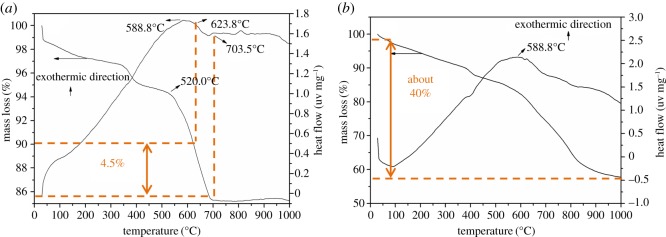
TG-DTA curves of Kuandian ores (*a*) and Kuandian ores/Na_2_CO_3_ (*b*).

According to the SEM and XRD results above, TG-DTA was carried out to investigate the thermal behaviours of Kuandian ores and to support the preliminary analysis of the mechanism for the sodium roasting process. As shown in figure 7*a*, the TG curve decreases slowly in the initial temperature (0–520°C). Meanwhile, owing to the removal of surface-absorbed water, the sample loses a small amount of weight. When the temperature reaches 588.8°C, the DTA curve presents an obvious exothermic peak, mainly because a large amount of Fe^2+^ in the sample is oxidized to Fe^3+^. Between 623.8°C and 703.5°C, a continuous and deep endothermal valley occurs in the DTA curve [24]. What is more, the TG curve decreases sharply at the same time and the weight loss rate reaches as high as about 4.5% in this region, the most convincing reason of which is that szaibelyite (Mg_2_(OH)[B_2_O_4_(OH)]) and antigorite (Mg_6_[Si_4_O_10_](OH)_8_) of the ores are dehydrated into suanite (Mg_2_B_2_O_5_) and forsterite (Mg_2_SiO_4_) [29,30]. As shown in figure 7*b*, when the temperature is raised to about 588.8°C, there is a significant exothermic peak caused by the oxidation of Fe^2+^ in the DTA curve, and then the roasting process presents a strong and sustained endothermic reaction. However, the significant heat-absorption valley appearing in the roasting process in figure 7*a* does not occur in the DTA curve and it is accompanied by a rapid sustained weight loss, with a weight loss rate of more than 40% at 1000°C, which is significantly more than that in the roasting process of Kuandian tailings. The reason lies in that on the one hand the dehydration decomposition reacts with the ascharite and antigorite during the roasting process of Kuangdian ores, and that on the other hand Na_2_CO_3_ absorbs a large amount of heat and evaporates the product CO_2_ during the sodium reaction between it and the dehydration decomposition reaction product of the ascharite and antigorite. It can be seen that compared with the single calcination of boron-bearing tailings, the mixed calcination of boron-bearing tailings and sodium carbonate may promote the sodium reaction, which is conducive to boron leaching and improves its leaching rate.

In combination with the analysis of the characterization results, this paper includes a preliminary analysis of the mechanism of the sodium roasting process. It can be learned that through sodium roasting, the soluble sodium borate produced by the reaction between Na_2_O (produced by the decomposition of Na_2_CO_3_) and Mg_2_B_2_O_5_ is beneficial to improve the leaching rate of boron-bearing tailings. The reaction equations related to the sodium roasting process are shown in (3.3) to (3.6). It can be seen that different factors have different effects on the sodium roasting process and the leaching rate. The roasting temperature mainly affects the decomposition of Na_2_CO_3_ and the degree of sodium roasting. When the roasting temperature is increased to 700°C, it fails to reach the decomposition temperature of Na_2_CO_3_ and Na_2_CO_3_ fails to decompose, so that the leaching rate is relatively low. When the roasting temperature reaches 750–950°C, Na_2_CO_3_ decomposition and sodium reaction occur. Besides, the amount of sodium carbonate mainly affects the types of substances involved in the sodium reaction during the roasting process. When one to four times the theoretical amount of Na_2_CO_3_ is added, the Na_2_O produced by the decomposition of Na_2_CO_3_ mainly reacts with the quartz (SiO_2_) in the samples. The amount of Na_2_CO_3_ is less than that required for the reaction with boron in the boron-bearing tailings, so that the leaching rate is low. When the amount of Na_2_CO_3_ is increased to five times or six times the theoretical amount, the amount of Na_2_CO_3_ is enough for the sodium reaction, so that the leaching rate is high. When the amount of Na_2_CO_3_ is five times the theoretical amount, Na_2_O (produced by the decomposition of Na_2_CO_3_) mainly reacts with Mg_2_B_2_O_5_ to form Na_2_B_4_O_7_ which is easier to be leached. Nevertheless, when the amount of Na_2_CO_3_ is six times the theoretical amount, Na_2_O (produced by the decomposition of excessive Na_2_CO_3_) mainly reacts with Mg_2_SiO_4_ and Mg_2_B_2_O_5_, which produce, respectively, Na_4_Mg_2_Si_3_O_10_ and NaBO_2_. Moreover, with the increase of the roasting temperature, the sodium reaction proceeds more fully and the leaching rate is gradually improved. The roasting time mainly affects the degree of sodium reaction during the sodium roasting process. If the roasting time is too short, Na_2_CO_3_ cannot fully contact and react with the boron in the boron-bearing tailings, resulting in lower leaching rate, but when the roasting time is prolonged, the sodium reaction proceeds more fully, which therefore is beneficial to improve the leaching rate.

3.1 $$\text{M}{\text{g}}_{6}\text{S}{\text{i}}_{4}{\text{O}}_{10}{\left(\text{OH}\right)}_{8}\to 3\text{M}{\text{g}}_{2}\text{Si}{\text{O}}_{4}+\text{Si}{\text{O}}_{2}+4{\text{H}}_{2}\text{O, }$$

3.2 $$\text{M}{\text{g}}_{2}{\text{B}}_{2}{\text{O}}_{4}{\left(\text{OH}\right)}_{2}\to \text{M}{\text{g}}_{2}{\text{B}}_{2}{\text{O}}_{5}+{\text{H}}_{2}\text{O, }$$

3.3 $$\text{N}{\text{a}}_{2}\text{C}{\text{O}}_{3}\to \text{N}{\text{a}}_{2}\text{O}+\text{C}{\text{O}}_{2},$$

3.4 $$2\text{M}{\text{g}}_{2}{\text{B}}_{2}{\text{O}}_{5}+\text{N}{\text{a}}_{2}\text{O}\to \text{N}{\text{a}}_{2}{\text{B}}_{4}{\text{O}}_{7}+4\text{MgO, }$$

3.5 $$2\text{N}{\text{a}}_{2}\text{O}+3\text{M}{\text{g}}_{2}\text{Si}{\text{O}}_{4}\to \text{N}{\text{a}}_{4}\text{M}{\text{g}}_{2}\text{S}{\text{i}}_{3}{\text{O}}_{10}+4\text{MgO}$$

3.6 $$\;\text{and}\;\text{N}{\text{a}}_{2}\text{O}+\text{M}{\text{g}}_{2}{\text{B}}_{2}{\text{O}}_{5}\to 2\text{NaB}{\text{O}}_{2}+2\text{MgO}.$$

## 4. Conclusion

The leaching rate of boron-bearing tailings can reach 86.78% under the optimum conditions where the Na_2_CO_3_ amount is five times the theoretical amount, the roasting temperature is 950°C and the roasting time is 2 h. When the roasting temperature is 700–950°C, the leaching rate of boron-bearing tailings increases with the increase of roasting temperature; and with the increase of the amount of Na_2_CO_3_, the leaching rate increases first and then decreases, and the optimum amount of Na_2_CO_3_ is five times the theoretical amount. When the roasting time is 0.5–3 h, with the prolonging of roasting time, the leaching rate increases first and then decreases, and the optimum roasting time is 2 h. Furthermore, sodium roasting can significantly improve the leaching rate of boron-bearing tailings. The main reason for the increase of leaching rate is that Na_2_O (produced by the decomposition of Na_2_CO_3_ through sodium roasting) reacts with the boron in boron-bearing tailings to form soluble sodium borate. This result provides a guideline for the efficient comprehensive use of boron-bearing tailings and the development of boron industry.

## Data accessibility

This article does not contain any additional data.
